# Endocrine-Disrupting Chemicals’ (EDCs) Effects on Tumour Microenvironment and Cancer Progression: Emerging Contribution of RACK1

**DOI:** 10.3390/ijms21239229

**Published:** 2020-12-03

**Authors:** Erica Buoso, Mirco Masi, Marco Racchi, Emanuela Corsini

**Affiliations:** 1 Dipartimento di Scienze del Farmaco, Università Degli Studi di Pavia, Viale Taramelli 12/14, 27100 Pavia, Italy; mirco.masi@iusspavia.it (M.M.); racchi@unipv.it (M.R.); 2 Classe di Scienze Umane e della Vita (SUV), Scuola Universitaria Superiore IUSS, Piazza della Vittoria 15, 27100 Pavia, Italy; 3 Laboratory of Toxicology, Dipartimento di Scienze Politiche ed Ambientali, Università Degli Studi di Milano, Via Balzaretti 9, 20133 Milano, Italy; emanuela.corsini@unimi.it

**Keywords:** cancer, endocrine disruptors, tumour microenvironment, signal transduction, RACK1, immune system, EMT, ER, cytokine release, inflammation

## Abstract

Endocrine disruptors (EDCs) can display estrogenic and androgenic effects, and their exposure has been linked to increased cancer risk. EDCs have been shown to directly affect cancer cell regulation and progression, but their influence on tumour microenvironment is still not completely elucidated. In this context, the signalling hub protein RACK1 (Receptor for Activated C Kinase 1) could represent a nexus between cancer and the immune system due to its roles in cancer progression and innate immune activation. Since RACK1 is a relevant EDCs target that responds to steroid-active compounds, it could be considered a molecular bridge between the endocrine-regulated tumour microenvironment and the innate immune system. We provide an analysis of immunomodulatory and cancer-promoting effects of different EDCs in shaping tumour microenvironment, with a final focus on the scaffold protein RACK1 as a pivotal molecular player due to its dual role in immune and cancer contexts.

## 1. Introduction

Steroid hormones can interact with specific receptors, orchestrating a vast set of physiological functions, including growth, development, reproduction, energy imbalance, metabolism, immunity and behaviour [1]. These hormones derive from cholesterol and can be divided into corticosteroids (glucocorticoids and mineralocorticoids) and sex steroids (androgens, oestrogens, and progestogens). Steroid hormones are present in body fluids and act at nanomolar concentrations to ensure a continual dialogue between the endocrine system and the other two main communication systems of the body, the nervous system and the immune system. Any alteration of the endocrine system may also affect these other two systems [1]. In this regard, certain man-made and natural chemicals, known as endocrine-disrupting chemicals (EDCs), have been reported to affect the endocrine system functions, interfering with hormone action, thereby increasing the risk of adverse health outcomes [2] including reproductive impairment [3,4,5], cognitive deficits [6,7,8], metabolic diseases and disorders [9,10] and various tumours, mainly breast (BC) and prostate cancer (PC) [11,12,13,14].

Human exposure to EDCs can occur via ingestion (food, dust and water), via inhalation (gases and particles in the air) and through the skin. EDCs can be found in food contact materials, cosmetics, consumer goods (including furnishings, cleaning products), toys, as well as in drinking water. Moreover, EDCs can act on similar or different pathways displaying cumulative or synergistic effects. These effects can be observed in different temporal windows (i.e., pre- and postnatal life, puberty and adulthood), with adverse effects in both the short- and long-term [15]. Hence, the deleterious effects of EDCs represent a health issue due to their potency, constant and universal human exposure [16].

EDCs are known to display hormonal features, including oestrogen and androgen activities, and they have been correlated with increased tumour risk considering their effects on cancer progression [11,12,13,14]. The tumour microenvironment plays an important role in establishing the cancer phenotype by interacting with the immune system. The role of EDCs in modulating the tumour microenvironment has not been elucidated, but is of pivotal interest. In this regard, the scaffold protein Receptor for Activated C Kinase 1 (RACK1) is an EDC target in the immune context [17,18,19,20] and an important molecular player for cancer progression (reviewed in [21]). Therefore, EDCs-mediated RACK1 regulation in both contexts could be central to understand the role of endocrine-mediated microenvironment regulation and to link innate immune activation with cancer progression through RACK1. In this review, we discussed RACK1 dual role as a possible molecular bridge for cell response to EDCs in immune and cancer system.

## 2. Concepts of Endocrine Disruption

### 2.1. EDCs: Definition and Characterisation

The definition of EDCs proposed by the World Health Organisation (WHO) and International Programme on Chemical Safety (IPCS) in 2002 [22] is now widely accepted scientifically: “*An endocrine disrupter is an exogenous substance or mixture that alters function(s) of the endocrine system and consequently causes adverse health effects in an intact organism, or its progeny or (sub)populations*” [22]. To provide a uniform basis for searching, organising and evaluating mechanistic evidence to support the identification of EDCs, a new approach based on ten key characteristics has been recently proposed. These include EDCs’ chemical interactions with hormone receptors (HRs) (binding, agonism and/or antagonism), HRs’ epigenetic and expression modifications, alterations of hormone signal transduction, synthesis, metabolism, transport, distribution and clearance, all contributing to alter the cellular fate of hormone-responsive cells [2]. In this regard, since endocrine signalling is involved in controlling all aspects of pre- and post-natal development, alterations of endocrine system may play a role in increasing susceptibility to several diseases and disorders, ranging from congenital malformations, metabolic disorders, fecundity, neurological disorders, cardiovascular disease and hormonally sensitive cancers including BC and PC [23]. Notably, as for endogenous responses, EDCs display a nonlinear, non-monotonic dose-response indicating that low exposure levels could show stronger effects compared to higher exposures [24]. This EDC effect can be ascribed to different molecular explanations, including different tissue sensitivity to EDCs [25], receptor desensitisation or internalisation, negative feedback loops. Altogether, these considerations finally suggest that EDCs functional characterisation needs an elevated number of doses to identify safe thresholds.

**Figure 1:**
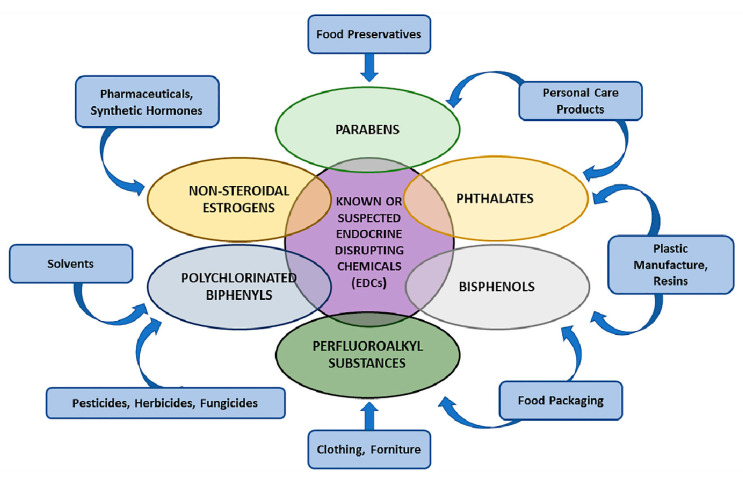
Most known EDCs’ chemical features and their classification. Suspected and known chemicals, having the potential to interfere with the endocrine system, are present in a variety of sources and result in human adverse health effects (see text for details).

EDCs can be classified based on their chemical features (e.g., non-steroidal oestrogens, parabens, phthalates, bisphenols, perfluoroalkyl substances (PFASs), polybrominated diphenyl ethers (PBDEs), polychlorinated biphenyls (PCBs)), adverse health effects or on the source of exposure (e.g., food contact materials, diet, cosmetics and personal care products, pharmaceutics, cleaning products and pesticides) [2] (Figure 1).

### 2.2. EDCs’ Molecular Mechanisms: From Dichlorodiphenyltrichloroethane (DTT) and Diethylstilbestrol (DES) as Proof of Concept to Other Sustances

Among the most known and studied EDCs, the insecticide DTT and synthetic non-steroidal oestrogen DES have been pivotal for the discovery of endocrine disruption. In this regard, DDT food contamination has been shown to negatively affect human health as demonstrated by the increase BC risk in females exposed to high DDT levels in utero [26]. Experimental data on the main metabolite of DDT, dichlorodiphenyldichloroethylene (DDE), provided direct evidence for immunosuppression resulting in the increased incidence of infectious diseases in DDT-exposed individuals [27,28]. DES has been reported to be the primary causative agent in clear cell carcinomas, reproductive disorders, infertility and spontaneous abortion in daughters of women exposed to DES [29]. In addition, DES exposure has also been correlated with obesity [30] and increased risk of prostate and testicular cancer [31]. While DDT and DDE effects are correlated with their weak and strong AR antagonist profile respectively, DES is characterised by an oestrogenic activity displayed by its binding to oestrogen receptor (ER) [18,19] change.

A wide range of other substances, mostly man-made, are known or suspected to cause endocrine disruption. Several studies have identified a variety of EDCs molecular actions and chronic diseases associated with their exposure, representing a serious hazard for the population and the environment. In the following sections, the main EDCs classes and their molecular profile are discussed [16].

Bisphenols (BPA, BPAF, BPS) are industrial chemicals used in the production of polycarbonate plastics for food and beverages and epoxy resin-coated metal products [32]. Diet is considered the main source of BPA exposure in humans and, while banned for children food packing in the EU, the same is not true for general food packaging, thus making BPA exposure still present for pregnant women and, consequently, foetuses [33].

At the molecular level, BPA can interact with oestrogen receptors alpha and beta (ERα and ERβ), orphan receptor human oestrogen-related receptor gamma (ERRγ), Peroxisome Proliferator-Activated Receptor gamma (PPARγ) receptor, androgen receptor (AR), glucocorticoid receptor (GR) and G protein-coupled oestrogen receptor (GPER) [34]. The elevated number of receptors and signalling pathways influenced by BPA correlates with the multiplicity of health and biologic parameters affected by very low doses of BPA [35]. Similarly, BPA analogues can bind to ERα and ER and activate oestrogen signalling pathway [34].

Strong evidence suggests that BPA exposure may display several adverse effects leading to a variety of diseases including metabolic disorders (e.g., type 2 diabetes) [36,37], impaired memory performance [38], neurodevelopment [39] altered reproductive processes [40,41] and cardiovascular functions [42,43].

Conversely, BPA analogues binding to AR could affect its signalling cascade, which may potentially lead to cancer [44,45,46], as discussed in the following section. Molecular dynamic (MD) simulations data show that multiple binding sites with different affinities are available on AR for BPA, BPAF, and BPS, thus explaining the distinct AR-related toxicity observed with bisphenol chemicals [47].

Parabens are a large class of esters of the *p*-hydroxybenzoic acid, widely used as preservatives in foods, drugs and cosmetics. The most common parabens used in cosmetics are methyl-, propyl-, ethyl- and butyl-paraben. Despite their estrogenic and anti-androgenic effects, which have been linked to infertility due to modulation of oestradiol concentrations [48,49], no direct links with cancers have been reported [32]. In 2006, the European Medicine Agency (EMA) concluded on the absence of sufficient clinical evidence of parabens adverse effects in humans [50], although more recently some epidemiological studies showed effects on post-natal growth and increased weight in boys [51,52] and positive and negative associations with thyroid (e.g., free thyroxine) and reproductive hormones (e.g., oestradiol and progesterone), respectively, in pregnant women [53]. Consequently, further studies on parabens should be considered due to their frequent human exposure [54], also considering their ability to modify BPA and oestradiol pharmacokinetics by inhibiting enzymes pivotal for their metabolism, such as several isoforms of glucuronosyltransferase (UGT), sulfotransferase (SULT) and cytochrome P450 (CYP) [49].

Phthalates are a major group of industrial chemicals called plasticisers, used to increase flexibility and hardiness in plastics. They have a variety of appliances and are employed in personal care products, pharmaceuticals, medical care products, detergents and cleaning agents [55]. Moreover, a part of human exposure also comes from eating and drinking foods in contact with phthalates-containing products [56] and, to a lesser extent, from air contaminated with phthalate particles or vapours [32]. The most commonly used phthalates include di-*n*-butyl phthalate (DBP), di-2-ethyl-hexyl phthalate (DEHP) and dimethyl-phthalate (DMP) and, since most phthalates are not firmly bound to their matrix (notably the case for DEHP [57]), they can be transferred to and contaminate other substances, making phthalate exposure in humans ubiquitous in the EU population [58,59,60]. At the molecular level, DEHP has been shown to interact with AR displaying anti-androgen effects, PPAR receptors and aryl-hydrocarbon receptor (AhR) [61] and, as also for DBP exposure [62], disrupt the thyroid axis by influencing thyroid hormone cellular uptake and distribution [63].

Several adverse health effects have been linked to phthalate exposure. DEHP has been associated with various reproductive disorders in males, including an increased risk of abnormal sperm formation [64], reduced anogenital distance due to DEHP anti-androgenic features [65], and altered testicular function from reduced testosterone and insulin-like-factor-3 levels [66]. Moreover, DEHP exposure has also been correlated with developmental neurotoxicity [67,68], increased oestrogen levels in pregnant women [69], increased risk of preterm birth [70] and developmental delay and autism spectrum disorders due to alterations of thyroid hormones after prenatal exposure [71].

Perfluoroalkyl substances (PFASs) are a category of industrial chemicals employed for different purposes, mostly associated with their oil and water repellent chemical features. PFASs’ environmental persistency varies depending on their chemical features, as recently assessed by EFSA, and the ones considered persistent environmental pollutants are now being dismissed [72]. Most common PFASs include those employed for canned food containers and surfactants for waterproof surfaces, such as perfluorononanoic acid (PFNA), perfluorohexane sulphonic acid (PFHxS), perfluorooctanoic acid (PFOA) and perfluorooctane sulfonic acid (PFOS) [73]. It has been demonstrated that PFASs can be transferred to food [74], and consumption of food from PFASs-treated containers is associated with higher PFASs levels and decreased amount of circulating thyroid hormones [75]. PFOA and PFOS are of particular concern due to their long half-life (from four to five years) in humans, the ability to cross the placenta and, consequently, their bioaccumulation. Challenges in molecular characterisation of PFASs arise due to their chemical complexity and their continually changing profiles in the human body, together with their very different metabolism between rodents and humans. Several effects associated with PFAS exposure have been linked to influences on thyroid axis [76,77,78,79], weight gain [80], PFOA-related autoimmune disease ulcerative colitis [81], liver function [82] and neurodevelopmental disorders after prenatal exposure [83]. In addition to the PFAS-mediated thyroid effects, alteration in immune response is correlated with PFASs levels [84,85]. Alteration in immune response included increased production of pro-inflammatory cytokines correlated with PFASs and other EDCs exposures in women during pregnancy and the postpartum period [86]. However, the involvement of endocrine mechanisms and the role of nuclear receptors expressed in human immune cells [87] still need to be completely elucidated.

## 3. Cancer Risk Linked to EDC Exposure

Strong evidence has accumulated on the implication of known and suspected EDCs in different types of cancer. A correlation between DES exposure and clear cell adenocarcinoma of the vagina was first reported in 1971 [88], but other particular cases include the association between chlordecone (a banned chlorinated pesticide) [89] and BPA [90] with increased PC risk, and the correlation of other EDCs, such as biocides and flame-retardants, with an increased incidence of papillary thyroid cancer [91,92,93]. Moreover, BPA has been strongly suggested to be a human carcinogen for BC and PC due to its tumour-promoting features [90], and PFOA, a member of one of the main EDC categories named PFASs, has been associated with increased testis and kidney cancer incidence in individuals exposed to industrially contaminated drinking water [94].

Since EDCs are exogenous chemicals and pollutants capable of altering the endocrine system by interfering with different aspects of hormone action and displaying epigenetic effects, the correlation between EDCs exposure and hormone-sensitive cancer types, in particular BC and PC, is of pivotal interest. Interference of different EDCs with ER [95] and AR [96] has been widely screened, but for other receptors such as GR potentially or known to be involved in the development and progression of hormone-related cancer types, further studies are required. In addition, EDCs’ interference with androgen-to-oestrogen converting enzyme aromatase—a cytochrome P450 involved in a variety of roles ranging from bone mineralisation, glucose homeostasis, ovarian follicle development, placenta and brain functions [97]—has been reported at both enzymatic and gene expression levels for different categories, including bisphenols, PCBs, phthalates and several pesticides [98].

In the following sections, the links between EDCs exposure with cancer in females and males, with a focus on BC and PC respectively, are discussed.

### 3.1. EDCs Associated with Hormone-Sensitive Cancers in Females and Males: Focus on BC and PC

Among female individuals, BC is one of the most common malignancies worldwide [99] and its aggressive nature is caused by abnormal regulation of cell proliferation and migration, contributing to tissue invasion and metastasis formation [100]. On the other hand, among male individuals, PC is the most common, non-dermatological epithelial malignant tumour in developed countries [101] and its aggressive nature has been investigated and correlated with important signalling pathways—mostly PI3K/Akt pathway-related—involved in proliferation, invasion, migration and cell survival [102,103,104,105]. These tumour-sustaining mechanisms are influenced by multiple endocrine-related pathways that can be deregulated after EDCs exposure and strong experimental and epidemiologic evidence supports the implication of hormonal-acting compounds in BC and PC incidence.

#### 3.1.1. Non-Steroidal Oestrogens (DES and Zearalenone)

In utero exposure to DES has been associated with increased BC incidence at puberty [106,107,108], and epidemiologic data correlated exposure to oestrogenic compounds during foetal development to increased risk of BC and PC [109,110,111]. In a recent study, in utero exposure to DES and BPA was linked to increased BC susceptibility due to increased collagen deposition and extracellular matrix density, which contribute to increase breast stiffness, and in turn correlated with a higher BC risk [112]. DES induces increased ERα-mediated gene expression of *CYP26A1* and *CYP26AB1*, which are responsible for metabolism and elimination of retinoic acid [113,114]. Changes in CYP metabolism due to DES could result in developmental toxicity as a result of epigenetic changes on DNA methylation [115,116] and acetylation [117]. In addition to the widely studied effects of DES, another non-steroidal oestrogen potentially implicated in BC and PC increased risk is the mycotoxin zearalenone (ZEA), which has been found to exert its effects on both ER and GPER [118]. Interestingly, it has been revealed that ZEA might display pro- and anti-proliferative potential on PC cells depending on its cellular concentration [119]. Epidemiologic evidence identified a potential role of ZEA and its metabolites α- and β-Zearalenol (α-ZOL, β-ZOL) in BC [120]. This observation is supported by further in vitro studies that reported ZEA, α- and β-ZOL to display growth-promoting effects in breast tissue by increasing protein synthesis and lipid metabolism, thus inducing a potential oestrogen positive BC progression [121]. Notably, ZEA has been shown to induce ERα-mediated migration and invasion of PC cells [122], while ERβ and NF-κB were shown to exert a protective role in PC cells against ZEA-induced oxidative stress [123].

#### 3.1.2. DDT

Epidemiological data reported a positive correlation between DDT, among other environmental pollutants, with BC risk [124,125], but not for PC. Indeed, intra-uterine DDT exposure has been identified as a putative BC risk factor [26] and positively associated with higher mammographic breast density, an intermediate marker of BC risk [126,127], whereas DDT exposure in early childhood may increase BC risk in adulthood [128]. Moreover, a prospective study on young women reported a significantly increased BC risk with increasing *p,p’*-DDT serum levels [129,130]. This is possibly due to the different actions of DDT metabolites, since isomers *p,p’*-DDT and *o,p’*-DDE display an oestrogenic activity. In contrast, *p,p’*-DDE is mainly anti-androgenic and has been suggested to accelerate tumour onset in BC mice model [131]. Interestingly, low-dose DDT exposure has been associated with increased aromatase activity and mRNA transcription, increased aromatase-induced 17β-estradiol (a breast carcinogen) biosynthesis and, ultimately, an increased ERα-mediated BC proliferation [132]. These data align with the association between DDT and other organochlorine pesticides in BC tissue specimens and the observed molecular dysfunction [133]. In addition, DDT exposure has been linked to differentially methylated regions involved in BC susceptibility, suggesting that prenatal DDT exposure may induce gene alterations with life-long consequences [134].

#### 3.1.3. Bisphenols

BPA can interact with different hormone nuclear and membrane receptors and is considered a possible BC risk factor due to its mammary cell growth-promoting properties [90]. Indeed, BPA exposure was observed to result in the development of pre-cancerous and cancerous lesions in mammary glands of rodent models [135] and its perinatal exposure has been reported to induce long-term alterations in hormonal response, thus increasing BC development propensity [136]. In addition, repeated and chronic exposure to BPA has also been shown to play a role in BC progression with poor patient outcome [137], as suggested by gene expression data supporting increased cancer aggressiveness [138]. In 2020, it has been reported that in utero BPA exposure is associated with increased BC susceptibility and higher BC risk [112]. At a molecular level, these observations are supported by several experimental evidence, both in vitro and in vivo [135,136,137,138]. These include in vitro ERα-mediated effects of BPA and its analogues on the upregulation of genes involved in cell growth, migration, invasion and cancer development [139], and low doses BPA-induced phosphorylation of its functional non-genomic target Protein Kinase D1 (PKD1), which mediates cell proliferation and anchorage-independent growth in BC cells [140]. Moreover, BPA oncogenic potential regarding BC is also found in its ability to enhance GPER-induced cancerous phenotype [141] and focal adhesion assembly through focal adhesion kinase (FAK), Src and ERK2 in a triple-negative BC model [142]. Others have shown that exposure to xenoestrogens like BPA can increase adult prostate size and induce PC [143]. Indeed, environmental exposure to BPA increased prostate sensitivity to develop prostate intraepithelial hyperplasia (considered a pre-neoplastic lesion) following a second exposure in adulthood [144].

Bisphenol AF (BPAF), has replaced BPA in industrial settings but increasing amounts of data suggest an increased ER binding affinities than BPA. Therefore, BPAF promotes BC cell growth and progression, inducing endogenous transcription of oestrogen responsive genes through genomic and nongenomic pathways involving the ERα and ERK1/2 activation, respectively [145]. In this regard, BPAF promotes ER-positive BC cell proliferation by enhancing the crosstalk between the membrane glycoprotein amphiregulin (AREG) and tyrosine kinase receptor signalling [145]. Moreover, BPAF can trigger GPER signalling pathway leading to ERK and PI3K/Akt activation [146]. Similarly to BPA and BPAF, BPS exposure could be potentially linked to BC progression [147], but further investigations are needed regarding BPAF and BPS involvement in PC development.

#### 3.1.4. Phthalates

Among other EDCs, phthalates may act on steroid biosynthesis and have been reported to cause reproductive toxicity in females [148]. DBP and DEHP have been shown to induce proliferative effects via the activation of PI3K/Akt signalling pathway, while also displaying oestrogenic effects at low concentrations [149].

Regarding BC, phthalates’ action as potential oncogenic compound is still debated, and it is necessary to plan further investigations [150]. Although the correlation between phthalates’ exposure and PC development could potentially involve ERK5 and p38 mitogen-activated protein kinase (p38 MAPK) signalling, the role of phthalates in PC has been rarely reported [151] except for obese men exposed to DEHP and other phthalates [152].

#### 3.1.5. PFASs

Experimental evidence has shown that PFOS and PFOA, despite not possessing oestrogenic activity, enhanced 17β-oestradiol effect on oestrogen-responsive gene expression, ERK1/2 activation and growth in a BC hormone-deprived in vitro model [153], thus promoting proliferation, migration and invasion potential in human breast epithelial cells [154,155]. Nested case-control studies reported controversial data on the correlation between PFASs exposure and BC risk [156,157]. Similarly to BC, the same epidemiologic considerations are valid for PC and further investigations are needed [158].

## 4. Tumour Microenvironment (TME) and EDCs

### 4.1. Tumour Microenvironment as Promoter of Cancer Progression

The tumour mass consists of a heterogeneous population of cancer cells together with different resident and infiltrating host cells, secreted factors and extracellular matrix proteins, collectively known as the tumour microenvironment (TME) [159]. The dynamic interactions of cancer cells with their microenvironment consisting of stromal cells including stromal fibroblasts, endothelial cells and immune cells like microglia, macrophages and lymphocytes and the non-cellular components of extracellular matrix (ECM) such as collagen, fibronectin and laminin [160,161] are essential to promote cancer cell progression and metastasis [162]. Indeed, this intercellular crosstalk consists of a composite network of soluble factors (e.g., ECM remodelling enzymes, growth factors, cytokines, chemokines and inflammatory mediators), ECM, cell components and new emerging entities, such as exosomes, cell-free DNA (cfDNA), circulating tumour cells (CTCs) and apoptotic bodies [163,164]. The reciprocal cell–cell and cell–ECM interactions and the tumour cell hijacking of non-malignant cells force stromal cells to lose their function and acquire new phenotypes that promote the development and invasion of tumour cells [162], making the role of TME pivotal in favouring carcinogenesis and loss of tissue integrity [165,166]. Since tumour development is highly influenced by microenvironment dynamics, understanding how the different TME components potentially affect cancer progression is of central interest.

Among all tumour cells interactors in TME—which also include multifunctional pericytes involved in angiogenesis and tumorigenesis [167,168], tumour endothelial cells that support primary tumour growth [169] and cancer-associated fibroblast (CAFs) that produce ECM proteins for immunosuppression, recruit immunosuppressive cells and support tumour cells proliferation [170,171,172]—tumour-associated macrophages (TAMs) play a pivotal role as cellular components of the immune system. TAMs are key TME elements capable of affecting cancer cell behaviour [173] through migration-stimulating factors that favour tumour cell motility, metastasisation [174] and enhance cancer cell stemness by promoting Epithelial-Mesenchymal transition (EMT) [175,176]. In addition, modification of ECM composition and organisation (mostly performed by CAFs [177,178]) can also influence and promote tumour phenotype and metastasis formation when stiffness/rigidity, tension and molecular density are altered [179].

### 4.2. Immune System in TME and Its Tumour-Associated Macrophages

An important role in TME regulation is held by the host immune system, which has been reported to be involved in controlling development and progression of the tumour [180]. Indeed, during tumour development, cancer cells become resistant to the innate immune response and impair the adaptative response [181,182,183]. Cytotoxic CD8^+^ memory T cells, a common type of T lymphocytes in the TME, are capable of killing tumour cells [184] through the recognition of tumour-specific antigens and the consequent triggered, tri-phasic pathway immune response [185]. CD8^+^ T cells in the TME are supported by CD4^+^ T helper 1 cells (Th1), that release interleukin-2 (IL-2) and interferon-gamma (IFN-γ) [183] and Th2 cells-producing IL-4, IL-5 and IL-13 to support B cell response [181,186]. However, other immune cell populations can favour cancer progression by altering TME. In this regard, Th17 cells at TME level release IL-17A, IL-17F, IL-21 and IL-22 with antimicrobial action that favours tissue inflammation and promote tumour growth [185,186]. B lymphocytes in TME have been shown to play pivotal roles in regulating cancer cell proliferation and survival, induce chemoresistance and immune escape [164], and have also been linked to cancer-induced immunosuppression by initiating TGF-β-dependent conversion of FoxP3^+^ cells that contribute to tumour metastasisation [187,188]. CAFs have been reported to favour cancer cell proliferation by supporting metastatic site growth [189,190] and secreting fibroblast secreted protein-1 (FSP1) and other cytokines involved in initiating metastasisation in different cancer types, including BC [190,191].

A pivotal role in determining the importance of TME in cancer development and progression is held by TAMs, which support cancer cell invasion and clonal expansion by favouring tissue remodelling (e.g., Epidermal Growth Factor, EGF; matrix metalloproteinase-2 and 9, MMP2, MMP9; Membrane type 1-matrix metalloproteinase, MT1-MMP) and pro-inflammatory molecules (e.g., IL-1β, TNF-α and C-X-C motif chemokine ligand 10 (CXCL10) [192]. Moreover, TAMs immune functions can facilitate tumour cell proliferation, migration and survival through cancer cell-induced release of specific growth factors and cytokines [193], while expression of vascular cell adhesion molecule 1 (V-CAM1) allows TAMs proliferation upon differentiation into inflammatory monocytes [194].

### 4.3. EDCs as Landscape Shapers in BC- and PC-Associated TME

It is noteworthy that EDCs can affect oestrogen signalling cascades by promoting a crosstalk between BC cells and fibroblasts, which have been shown, for example, to increase aromatase expression or secrete several growth factors able to trigger rapid oestrogen-related pathways in cancer cells [195], ultimately contributing to cancer cell progression, invasion and metastasis formation. Indeed, EDCs in stromal cells are capable of mediating cellular differentiation and survival mechanisms [196,197,198], although their effect on ERα-, ERβ-, and GPER-related functions and expression in the stromal components still needs to be demonstrated.

BPS has been shown to exert oestrogenic activity on stromal and stem cells in BC context [199,200] and to enhance lipid accumulation through an ER-mediated mechanism [200], while BPA is capable of promoting cell survival after DNA damage [198] and driving adipocyte differentiation through its ERR-γ activity [201,202]. Moreover, DDT and its metabolite DDE have been reported to induce an oestrogenic microenvironment in breast adipose tissue [98], which may support cancer phenotype establishment. In this regard, oestrogen-mediated signalling was observed to display an important impact on ECM matrix composition [203]. Breast tumourigenesis and malignancy is tightly linked with differential collagen crosslinking and clustered integrin-mediated formation of focal adhesion, resulting in increased tumour stiffness [204]. In turn, integrin clustering and consequent increased tumour rigidity have been shown to promote cancer growth by enhancing growth factor signalling and focal adhesion assembly [205]. Interestingly, BPA has been reported to increase collagen fibre content and cell proliferation [206], suggesting that EDCs can influence matrix remodelling in a pro-tumorigenic manner. Indeed, high collagen content has been associated with increased carcinogenesis and oestrogenic signalling was observed to modulate collagen, integrin, MMP2 and MMP9 expression in BC context [207,208,209], supporting the hypothesis that environmental EDCs exposure may play a mechano-transductive role in oncogenic ECM remodelling and cell-ECM crosstalk, especially in TME context [210].

Regarding PC, EDC exposure has been reported to possibly reprogram or transform adult prostate progenitor cells favouring their tumour-initiating capacity through ER signalling pathways. In this regard, BPA has been shown to display carcinogenic potential by inducing PC cell proliferation, differentiation defects of the adult epithelium, thus predisposing to prostate dysplasia. Moreover, BPA has also been observed to induce epigenetic mechanisms leading to PC cell reprogram. These considerations highlight the potential to provide evidence for an effect of EDCs exposure on human prostate [211]. However, literature data lack studies on PC-related TME involvement and the possible role of EDCs in this context.

In light of the effects of different classes of EDCs on BC and PC development and progression discussed in the previous section, investigating EDCs role in TME functional alteration may allow a deeper understanding of EDCs effects not only on the tumour stromal component (i.e., fibroblasts) and their consequent involvement in cancer initiation and progress [212], but also on the immune system cells that are present within the TME and that could play an important role in establishing tumour development and progression. In this regard, accumulating evidence suggests that EDCs can affect the immune system and induce functional alteration in the immune response—both innate and adaptive [213,214]—potentially resulting in adverse reactions, immunosuppression, autoimmunity and enhanced immunostimulation [215]. Notably, TNFα, a pleiotropic cytokine involved in body’s inflammatory response, is mainly produced by monocytes and macrophages after phthalates exposure [216]. In addition, EDCs can modulate production and release of several pro-inflammatory interleukins, including IL-1β, IL-6 and IL-8 [216]. Moreover, enhanced DEHP-induced chemokine production [217] and increased BPA-mediated monocyte chemotactic protein (MCP-1 also known as CCL2) were observed [218]. EDCs have also been reported to hamper neutrophils function (e.g., DDT-induced decreased chemotaxis, phagocytosis adhesion and oxygen-dependent killing) [28] as wells as affect maturation of dendritic cells (DCs). In this regard, BPA decreases DCs endocytic ability [219] and increases release of IL-5, IL-10 and IL-13 upon TNFα [220]. Furthermore, DEHP and BPAF can suppress ERK1/2 and NF-κB activation in DCs, affecting their maturation [219]. In lymphocytes context, DDT decreases NF-κB expression, ultimately leading to reduced IL-2 production [221].

EDCs can display their effect on the immune system through several mechanisms that mainly involve estrogenic receptors ER, ERRs, PPARγ and GPER, thyroid receptors and AhR [222]. In this regard, phthalates and BPA have been shown to induce alteration of cytokines levels through ER-mediated signalling. Moreover, BPA alters ER and ERRs expression in a dose and sex-specific manner. Indeed, BPA was observed to influence T-cell function through ERRα expression modulation, suggesting that EDCs may exert their immunomodulatory activities by targeting ERRs [223]. In addition, BPA acts as antagonist for PPARγ, an adipocyte-specific receptor involved in adipogenesis with typically anti-inflammatory effects, indicating that EDCs can promote a pro-inflammatory phenotype in immune cells [223].

Altogether, these observations highlight the importance of a deeper understanding of EDCs immunomodulatory effect in TME context, based on the consideration that alteration of released cytokine pattern and other immune-related features can affect cancer development and progression. In this regard, investigating molecular EDC targets in both contexts is of pivotal interest for a better characterisation of the crosstalk between the different TME components able to influence reciprocally.

## 5. RACK1 as a Possible Target of EDCs

Accumulating evidence indicates that EDCs can significantly affect the immune response in human and wildlife [2]. Many EDCs can interfere with the immune system displaying immunosuppressive properties, as well as enhanced autoimmune reactions and increased inflammation [215]. Indeed, steroid hormones can influence the initiation of immune response and the maintenance of peripheral tolerance of self-antigens, indicating that an altered regulation of hormonal action can lead to immunotoxicity [16]. Several mechanisms have been associated with oestrogen-induced immunotoxicity, including upregulation of co-stimulatory molecules and pro-inflammatory cytokines [16]. Conversely, androgens and corticosteroids are reported to significantly modulate the immune response resulting in anti-inflammatory and immunosuppressive actions [16]. Moreover, it is particularly interesting to consider the hormone dehydroepiandrosterone (DHEA), since it is a precursor of androgens and oestrogens and exerts anti-glucocorticoid effects in several systems, especially in the immune system [224,225,226], where DHEA counteracts cortisol effects. This interaction of DHEA and glucocorticoids (GCs) in the immune system is partly related to the effect of these hormones on the expression of the protein Receptor for Activated C Kinase 1 (RACK1) [227].

RACK1 is a member of the tryptophan-aspartate repeat (WD-repeat) family of proteins, originally found to act as a shuttling protein for activated protein kinase C βII (PKCβII) and other PKC isoforms [228]. Indeed, a defective PKCβII translocation due to age-associated RACK1 decline has been described in different immune cells [229,230], highlighting that reduced expression of RACK1 is related to a significant decrease in immune cells functionality, including response to influenza vaccination [231] and cytokines production [232,233,234]. Literature data report that DHEA can restore the age-associated decline of RACK-1 expression and immune functions both in vitro and in vivo [233,234,235]. The key mechanism of DHEA positive effect on RACK1 expression and monocyte activation is the conversion of DHEA to active androgens, which act via AR, justifying DHEA anti-glucocorticoid action on RACK1 expression described in the immune context [20,227]. Indeed, the bioinformatics analysis of the RACK1 promoter revealed the presence of a glucocorticoid response element (GRE) consensus sequence identified at the nucleotide position −186/−165 relative to the transcription start site [20]. Accordingly, physiological concentrations of cortisol can down-regulate RACK1 expression by inhibiting its gene promoter activity [227]. Further evidence demonstrated that other corticosteroids such as betamethasone, budesonide, methylprednisolone, prednisone and prednisolone can target RACK1, supporting the notion that this protein is an important target of corticosteroid-induced anti-inflammatory effects [236]. In this regard, DHEA counteracts GCs action on RACK1 expression by interfering with GR splicing [237]. The human GR gene (*NR3C1*) is expressed in several isoforms and the most representative are GRα and GRβ. GRα mediates most of the known glucocorticoid actions, while the GRβ isoform lacks the ligand-binding domain and have a dominant negative effect on GRα [238]. DHEA can induce the up-regulation of GR mRNA, which is preferentially spliced toward the β isoform due to an increase in expression of the splicing factor Serine/arginine (SR)-Rich Splicing Factors 9 (SRSF9). On the other hand, cortisol upregulates SRSF3, the splicing factor promoting GRα isoform. Therefore, DHEA and cortisol act on RACK1 expression by modulating SRSF9 and SRSF3 in a different way, highlighting that the anti-glucocorticoid effect of DHEA is exerted by modulating GRβ expression and ultimately antagonising the GRα effect on RACK1 promoter [237]. Moreover, DHEA-induced RACK1 expression and immune cell activation is related to DHEA conversion to androgens, which act through their binding to RACK1 promoter [20]. Indeed, approximately one-half of the AR cistrome overlaps with that of GR because the DNA-binding domain (DBD) of androgen and glucocorticoid receptor is highly conserved. Hence, they recognise a response element usually described as a canonical androgen/glucocorticoid response element (ARE/GRE) characterised by a well-conserved 5′-hexamer and a less stringent sequence requirement for the 3′-hexamer. Therefore, the GRE sequence described in the *RACK1* gene promoter is also a *cis*-regulatory target of AR [17,18,20] as further confirmed by nandrolone induced-RACK1 expression and the immune response [18]. These data demonstrate that a complex hormonal balance, between cortisol and androgens, can regulate RACK1 expression and immune activation [20] thus supporting RACK1 as a possible target of EDCs with a consequent modulation of innate immune functionality and cell proliferation [16,18]. According to these data, it has been demonstrated that *p,p’*-DDT and its main metabolite *p,p’*-DDE, a weak and strong AR antagonist respectively, negatively modulate RACK1 expression and the innate immune response thus reducing LPS-induced IL-8 and TNF-α production and CD86 expression [18] consistent with their described immunosuppressive effects [239,240]. Moreover, *p,p’*-DDE exerts a stronger repression effect than *p’p’*-DDT, according to its higher affinity for AR [18]. These results demonstrate that RACK1 is a bridge between the endocrine system and the innate immune and it can be regulated in an opposite way by agonists and antagonists of AR, thus supporting RACK1 as a target of EDCs.

RACK1 expression and immune functionality can be also modulated by the endogenous hormone 17β-oestradiol which up-regulates RACK1 expression levels and increases response to LPS [19]. Based on this observation, RACK1 expression and its related immunological implications were investigated after treatment with the synthetic oestrogen DES or the oestrogen-active compound of natural origin, ZEA. All compounds increased RACK1 transcriptional activity, which paralleled an increase in LPS-induced IL-8, TNF-α production, and CD86 expression [19], which we previously demonstrated to be dependent on RACK1/PKCβ activation [241]. These oestrogen-active compounds effects are mediated by the plasma membrane GPER and AR activation, as flutamide can completely prevent DES-induced RACK1 expression [19]. Hence, exposure to oestrogen-active compounds is associated with increased immunostimulation, which should be considered indicative of immunotoxicity [19].

Nowadays, RACK1 is known to be involved not only in the immune response but also acts as a signalling hub, facilitating the cross-talk between several pathways involved in various biological events such as neuronal activity [242,243] and cancer progression [19]. Indeed, RACK1 is aberrantly expressed in several cancer types [244], including BC [21,245]. In this regard, RACK1 has been suggested as a possible BC biomarker [246,247] since it has been directly correlated with increased proliferation rate, migration and invasion of BC cells both in vitro and in vivo [248,249,250] and emerged as superior predictor of BC prognosis [250]. Moreover, RACK1 has been revealed as strong regulator of cell cycle progression and apoptosis [251,252] according to in vivo data showing its role in paclitaxel chemoresistance in BC cells [252].

Literature data here presented strongly suggest a tight correlation between EDCs exposure and their involvement in cancer development and progression and, on the other hand, in alterations of the immune system through their activity on TME and cellular signalling cascades. In this regard, the identification of RACK1 as a possible EDC target in the immune context and, at the same time, its importance in tumour progression may indicate that RACK1 could play a dual role in BC- and PC-associated TME establishment and in modification of the immune response, particularly related to xeno-oestrogenic EDCs.

**Figure 2:**
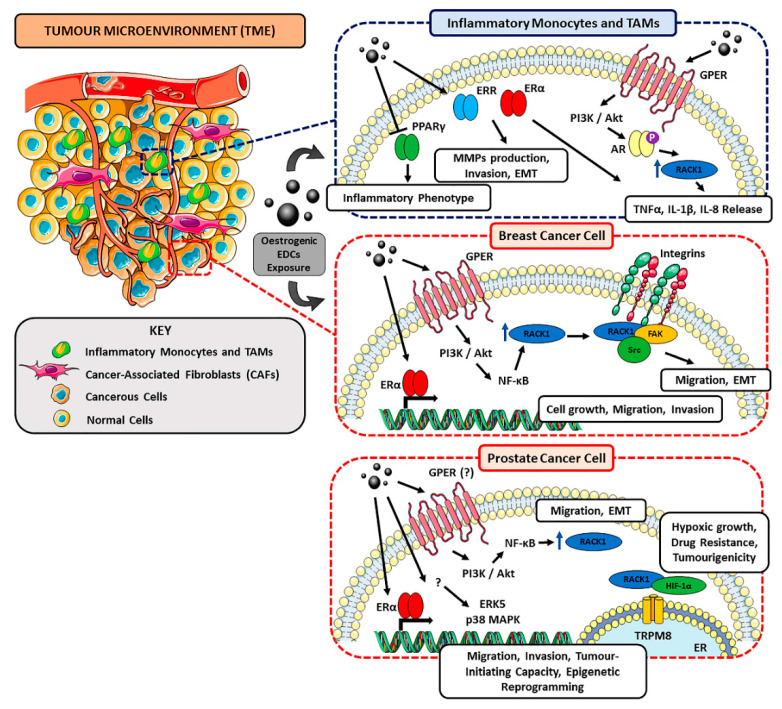
Schematic representation of the possible dual role of RACK1 in immune and breast/prostate cancer contexts in EDC-related TME and tumour progression. Oestrogenic EDCs (e.g., BPA, DES, ZEA) can promote the proliferation and migration of hormone-responsive and triple-negative BC cells where RACK1 appears to be involved due to its role in favouring proliferation and Epithelial-Mesenchymal Transition (EMT). Regarding prostate cancer, although RACK1 has been reported to promote PC cell proliferation, migration and invasion [256] and to mediate hypoxic growth, chemoresistance and tumorigenicity through TRPM8 channel and HIF-1α axis [257], most EDC-mediated mechanisms have still to be completely elucidated and further investigation is required. Altogether, oestrogenic EDCs can display cancer-promoting effects by inducing alterations in the immune system, including an increased release of pro-inflammatory cytokines and ECM-remodelling factors (see text for details).

Different EDCs, in particular DES, ZEA, and BPA, have been shown to activate ER-related pathways by binding different receptors (e.g., ERα, ERβ, GPER), thus inducing an oestrogenic TME that determines an aberrant immunostimulation through the upregulation of co-stimulatory molecules and pro-inflammatory cytokines. In parallel, they promote cancer phenotype through alterations of key cellular and tumour-related mechanisms and ECM remodelling. Noteworthy, BPA has been shown to activate FAK, Src and ERK2 kinases inducing focal adhesion (FAs) assembly to promote cancer cell migration in a triple-negative BC model [142,253]. This is particularly relevant in cancer context, since RACK1 has been demonstrated to directly bind FAK and this complex is recruited at focal adhesion level for cancer cell migration and invasion [250,254]. Indeed, RACK1 acts as a scaffold for phosphorylated FAK in response to integrin binding [255]. In this regard, integrin clustering is sufficient to induce FAK Tyr397 phosphorylation necessary for its activation. In turn, FAK binding to RACK1 generates a Src binding site, that furtherly promotes FAK-RACK1 complex formation and full FAK activation. Notably, increased ECM rigidity due to TAMs and CAFs-mediated changes in its composition can also induce integrin clustering and promote FAs assembly [205], highlighting TME’s pivotal role in cancer phenotype establishment. Furthermore, since oestrogenic EDCs are able to promote proliferation and migration of hormone-responsive BC cells (and, to a lesser extent, of PC cells), RACK1 appears to be involved in this context due to its role in favouring EMT, as suggested in other works. Since alterations of RACK1 expression in both in BC and PC cells have been reported to be mediated by NF-κB through PI3K/Akt signalling cascade, oestrogenic EDCs can mediate GPER activation leading to RACK1 overexpression, thus promoting the acquisition of RACK1 extra-ribosomal functions which, in turn, favours EMT. On the other hand, in a mesenchymal cellular context (e.g., MDA-MB-231 cell line), RACK1 has been proposed to display more structural functions [21], and oestrogen-like EDCs may mostly influence FAK-related RACK1 roles (Figure 2). In addition to their direct effects on both normal and cancer cells driving their conversion to tumour phenotype and favouring cancer-progression respectively, EDCs can also display cancer-promoting indirect effect by inducing alterations in the immune system. This in turn affects TME components, shaping TME cellular and molecular composition in a tumorigenesis sustaining way. As previously discussed, several EDCs have been correlated with monocytes and TAMs increased release of pro-inflammatory cytokines (TNFα, IL-1β, IL-6 and IL-8), enhanced MCP-1 production, reduced DCs maturation and endocytic ability and hampered neutrophils functions that lead to the establishment of a pro-inflammatory carcinogenic-promoting environment (Figure 2). In addition to these effects, TAMs favour cell invasion and clonal expansion through MMP2 and MMP9 release and support TME inflammation through their V-CAM1-mediated differentiation into inflammatory monocytes.

## 6. Conclusions

**Figure 3:**
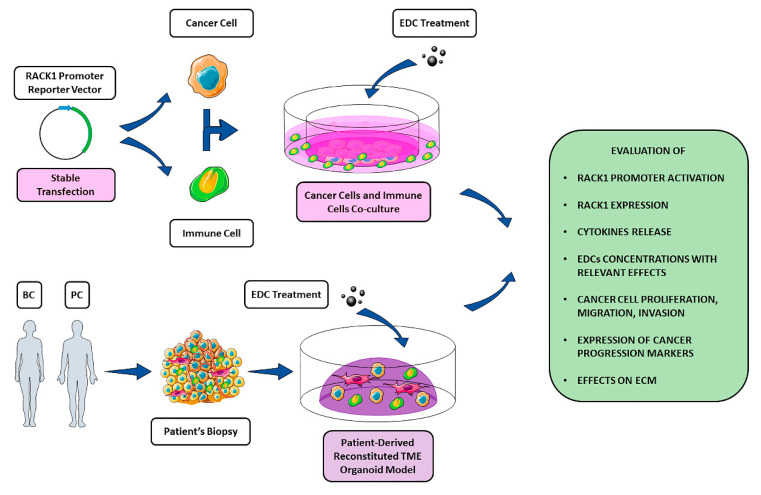
Schematic representation of in vitro and ex vivo strategies to investigate EDCs effects on TME. Due to its involvement in different key pathways in both cancer and immune context, RACK1 could be potentially used as a molecular tool to evaluate EDCs immune-correlated and tumorigenic effects. A panel of different evaluation targets is illustrated within the figure. While a strictly in vitro approach through cancer cells, immune cell co-cultures can benefit from genetic manipulation (i.e., stable transfection of both cell lines with a reporter construct containing RACK1 promoter region), an ex vivo strategy exploiting patient-derived organoid models for a better TME mimicking could allow the evaluation of EDC-mediated effects on the ECM.

In light of EDCs as drivers of TME-promoted cancer phenotype and RACK1 role as both EDCs target and signalling hub in the cancer context, we propose investigating RACK1 as a possible dual-role molecular player in TME. Indeed, RACK1 can favour EDC-mediated pro-inflammatory events in the immune context and, in parallel, promote cancer progression by binding key players necessary for cell proliferation, migration, invasion, metastasization and EMT (Figure 2). In addition, this could be particularly relevant for the development of in vitro and ex vivo strategies in which RACK1 could serve as investigation tool for evalutaing the effects of EDCs and other hormone-like substances on the immune functions and, simultaneously, their tumourigenic potential (Figure 3).
